# Neonatal and 3-month cerebrovascular oxygenation, stability, and extraction in congenital heart disease versus control infants

**DOI:** 10.1017/cts.2025.10106

**Published:** 2025-07-21

**Authors:** Nhu N. Tran, Jodie K. Votava-Smith, John C. Wood, Joanne Yip, Andrew Pham, Mary-Lynn Brecht, Panteha Hayati Rezvan, Anthony R. Colombo, Philippe Friedlich, Ken M. Brady, Bradley S. Peterson

**Affiliations:** 1 Institute for the Developing Mind, The Saban Research Institute, Children’s Hospital Los Angeles, Los Angeles, CA, USA; 2 Department of Pediatrics, Keck School of Medicine, University of Southern California, Los Angeles, CA, USA; 3 Division of Cardiology, Children’s Hospital Los Angeles, Los Angeles, CA, USA; 4 Keck School of Medicine, University of Southern California, Los Angeles, CA, USA; 5 Dornsife College of Letters, Arts and Sciences, University of Southern California, Los Angeles, CA, USA; 6 School of Nursing, University of California, Los Angeles, CA, USA; 7 Biostatistics and Data Management Core, The Saban Research Institute, Children’s Hospital Los Angeles, Los Angeles, CA, USA; 8 Department of Population and Public Health Sciences, Keck School of Medicine, University of Southern California, Los Angeles, CA, USA; 9 Fetal and Neonatal Institute, Division of Neonatology, Children’s Hospital Los Angeles, Los Angeles, CA, USA; 10 Lurie Children’s Hospital of Chicago, Anesthesiology and Pediatrics, Northwestern University Feinberg School of Medicine, Chicago, IL, USA; 11 Department of Psychiatry, Keck School of Medicine, University of Southern California, Los Angeles, CA, USA

**Keywords:** Cerebrovascular stability, cerebrovascular oxygenation, cerebral fractional tissue oxygen extraction, congenital heart disease, infants

## Abstract

**Objective:**

We compared indices for cerebrovascular health (i.e., physiological responses to tilts by measuring regional cerebral oxygenation [rcSO_2_], cerebrovascular stability, and cerebral fractional tissue oxygen extraction [FTOE]) in infants with congenital heart disease (CHD) versus healthy controls (HC) at neonatal and 3-month ages.

**Study design:**

Our cohort study included 101 neonates (52 CHD, 49 HC) and 108 infants at 3-months (45 CHD, 63 HC). We used an innovative and replicable evaluation tool to noninvasively and rapidly measure indices of cerebrovascular health. Changes in near infrared spectroscopy measures of rcSO_2_ after tilting (from supine to sitting, ∼150 values) assessed cerebrovascular stability. Mixed-effects regression models examined rcSO_2_ and FTOE differences between groups, and group-by-posture interactions, adjusting for postconceptional age, sex, ethnicity, and preductal systemic oxygenation (SpO_2_) at both ages.

**Results:**

Infants with CHD had significantly lower rcSO_2_ (13% at neonatal and 11% at 3-months, both *p* < 0.001), increased FTOE (∼0.14 points higher at neonatal and ∼ 0.09 points at 3-months, both *p* < 0.001), and reduced cerebrovascular stability compared with HC at both ages (both *p* < 0.001).

**Conclusions:**

CHD infants had persistently poorer indices of cerebrovascular health (i.e., lower rcSO_2_, increased FTOE, and reduced cerebrovascular stability) through the 3-month age compared to controls. Sustained cerebral hypoxia, reduced cerebrovascular stability, and increased FTOE may contribute to neurodevelopmental delays (NDDs) and could serve as early biomarkers for identifying infants at higher risk for NDD.

## Introduction

Children with congenital heart disease (CHD) face *five* times greater risk for neurodevelopmental impairments compared to their healthy peers [1], yet underlying causes remain unknown [2]. Potential physiological factors include dysregulated cerebrovascular health in children with CHD. We indexed cerebrovascular health with: (1) cerebrovascular autoregulation (CA), the brain’s homeostatic mechanism to regulate its blood flow [3], (2) regional cerebral oxygenation (rcSO_2_), and (3) cerebral fractional tissue oxygen extraction (FTOE) [4], which measures the brain’s oxygen consumption. Cerebral blood flow (CBF) relying on systemic blood pressure due to CA impairment may not fulfill oxygen demands, increasing the risk of hypoxic-ischemic injury [5]. Furthermore, increased FTOE may signal constant or elevated oxygen consumption with reduced or inadequate delivery. CHD neonates show increased FTOE and impaired CA preoperatively [6], intraoperatively [7], and immediately postoperatively [8], although it remains unclear whether these persist into early infancy.

We developed a novel measure (cerebrovascular stability) that uses repeated measures of changes in rcSO_2_ after tilting as a proxy for CA. Our method stimulates blood pressure fluctuations via tilting. We previously observed reduced cerebrovascular stability in CHD neonates compared to healthy controls (HC) using these methods [9], as rcSO_2_ declined in CHD neonates (suggesting impaired CA), but increased in HCs after tilting. Likewise, rcSO_2_ decreased when moving preterm [10] and healthy neonates in varying positions [11], but none have demonstrated differences in cerebrovascular stability and FTOE beyond the neonatal period. Therefore, our study assessed whether our indices for cerebrovascular health persists into the 3-month age. We expanded our dataset from a prior neonatal cohort and added data at the 3-month age [9]. We hypothesized that CHD infants would have poorer indices of cerebrovascular health (i.e., significantly lower rcSO_2_, reduced cerebrovascular stability, and increased FTOE than HCs at both ages. We compared these indices in single ventricle (SV) versus biventricular (BV) and cyanotic versus acyanotic cardiac defects.

## Methods

### Participants

Our 2-group (CHD and HCs), observational, and longitudinal cohort study examined indices of cerebrovascular health at neonatal and 3-month ages. We recruited CHD infants consecutively admitted to intensive care units and pregnant mothers from fetal cardiology clinics at Children’s Hospital Los Angeles (CHLA) between June 2018 and July 2023. HC were recruited from AltaMed within CHLA and Los Angeles community clinics. Parents provided written informed consent for all participating infants. All procedures adhered to ethical standards of the relevant national guidelines on human experimentation (Good Clinical Practice) and Helsinki Declaration of 1975, and was approved by the Institutional Review Boards of CHLA and AltaMed.

Inclusion criteria: (1) postnatal age of ≤14 days at the neonatal age and (2) gestational age ≥37 weeks at birth. CHD neonates were preoperative or pre-intervention requiring admission to CHLA. HCs had no major prenatal, delivery, or postnatal complications. Exclusion criteria: (1) congenital anomalies other than CHD, (2) major genetic abnormality, (3) intrauterine growth restriction, (4) maternal chorioamnionitis, (5) neurologic abnormalities such as seizures, (6) antibiotics use for a known infection, or (7), hemodynamic instability (dopamine ≥5mcg/kg/min) at the time of assessment. The CHD group had a cardiac intervention before their 3-month visit. We assessed infants within two weeks of birth or two weeks of turning 3-months old. Please refer to the Flow Diagram for sample details by age (Supplemental Fig. 1).

### Data collection

We placed an rcSO_2_ sensor on their forehead and connected to the monitor (INVOS 5100C Somanetics, Troy, MI), and connected a preductal arterial SpO_2_ sensor on the right hand to the Philips Intellivue MP70. Both monitors connected to a Bernoulli data aggregation system (Cardiopulmonary Corporation, Milford, CT). We swaddled the infants, ensuring a comfortable, calm state, and used a band to secure the head upright during tilts which prevented stress-induced intrathoracic pressure changes that potentially affect blood pressure. The examiner’s left hand supported the infant’s back and spine, while the right preserved head, neck, and chin alignment, ultimately minimizing slouching or abdominal compression.

We acquired data continuously for 2 minutes while the infant was in the supine (0°) posture, then tilted the infant to a sitting (90°) posture. Data acquisition continued for another 2 minutes in the sitting posture. We performed this process for 3 tilt cycles. If the infant became upset during data collection, we pacified the infant and restarted the procedure until 3 cycles were successfully completed.

### Measurements

We obtained demographic and clinical data, including medical history, cardiac anatomy and procedures, complications, comorbidities, and current medications from parent report and medical abstraction. Our outcome measures were rcSO_2_, cerebrovascular stability, and FTOE. Mean values were calculated from the last 2-minutes of the supine posture and the first 2-minutes of the sitting posture across three tilt replicates per participant. rcSO_2_ and SpO_2_ were recorded at 5-second intervals, ∼25 values in each posture for each of the 3 tilts, totaling ∼ 150 values obtained per participant at each age. We calculated FTOE for each participant at each 5-second interval using their rcSO_2_ and SpO_2_ with the formula: FTOE = ([SpO_2_-rcSO_2_]/SpO_2_).

### Statistical analyses

We performed statistical analyses using IBM SPSS Statistics version 27. A G×Power 3.1 analysis indicated a sample size of 46 would detect a large effect size (*d* = ∼0.6), with *α* = 0.05 and *β* = 0.80, when using linear mixed models for repeated measures comparing cerebrovascular stability and FTOE between groups [9,12].

We examined distributions, correlations, and scatterplots to detect outliers beyond 3 standard deviations. We checked outliers for entry errors and artifacts (e.g., movement), corrected mistakes, and excluded artifact-induced outliers from analyses. We included the few remaining outlier values as possible population representatives. Pearson correlation analyzed: (1) rcSO_2_ vs. FTOE and (2) rcSO_2_ vs. SpO_2_. Chi-square/Fisher’s exact tests and t-tests compared categorical variables and continuous variables between groups, respectively. We examined fit and assumptions for all models.

#### Hypothesis testing

Separate linear mixed models for repeated measures compared rcSO_2_, cerebrovascular stability, and FTOE between groups at both ages. Neonatal models included main effects for group and posture, group-by-posture interactions, and covariates of postconceptional age (the sum of gestational age at delivery and postnatal age), ethnicity, sex, and SpO_2_, with participants as a random factor. The 3-month models removed postconceptional age covariate based on literature suggesting minimal effect on rcSO_2_ and CBF by this age [6,13]. The group effect tested our hypothesis that CHD infants would have lower rcSO_2_ and higher FTOE than HCs. The group-by-posture interaction assessed whether cerebrovascular stability was reduced in CHD infants and if extraction was higher post-tilt.

#### Sensitivity analyses

We evaluated cerebrovascular stability and rcSO_2_ consistency by excluding the SpO_2_ from the models. Additionally, we ran the primary analyses for infants with visits at both ages, to compare the results. Lastly, we assessed the consistency of parameters for cerebrovascular stability when including postconceptional age as a covariate at the 3-month age.

#### Subgroup effects

Heart defects were classified at each age as SV versus BV and cyanotic (i.e., intracardiac defects that cause right to left shunting) versus acyanotic. Separate linear mixed models for repeated measures assessed whether rcSO_2_, cerebrovascular stability, and FTOE differed among defects at each age. The models included defect-by-posture interactions, main effects for ventricular type (SV vs. BV) or cyanosis (no vs. yes) and posture, and covariates of postconceptional age (only included in the neonatal model), sex, and SpO_2_.

#### Exploratory analyses

We determined whether posture effects observed for rcSO_2,_ and cerebrovascular stability extended to SpO_2_ by running the same models as listed in the hypothesis testing at both ages, but used SpO_2_ as the dependent variable. P-values were reported at a 2-sided significance level of 0.05, without multiple comparison adjustments for post hoc and exploratory analyses.

## Results

### Descriptive statistics

**Figure 1.**
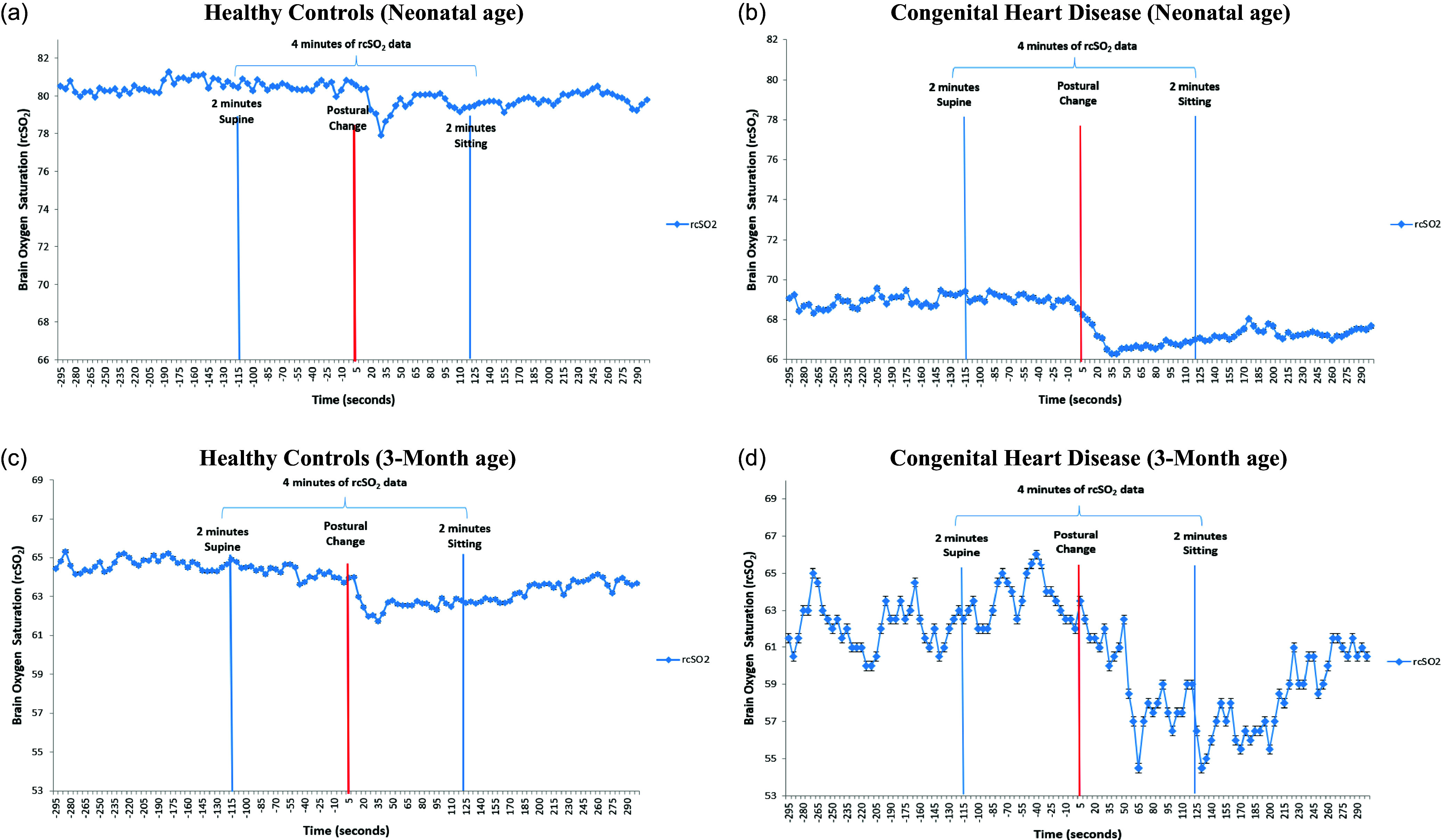
We display a time series of all rcSO_2_ values averaged at each time point across all infants in each group at the neonatal (a–b) and the 3-month ages (c–d). (a) Healthy controls (Neonatal age). (b) Congenital heart disease (Neonatal age). (c) Healthy controls (3-month age). (d) Congenital heart disease (3-month age).

**Figure 2.**
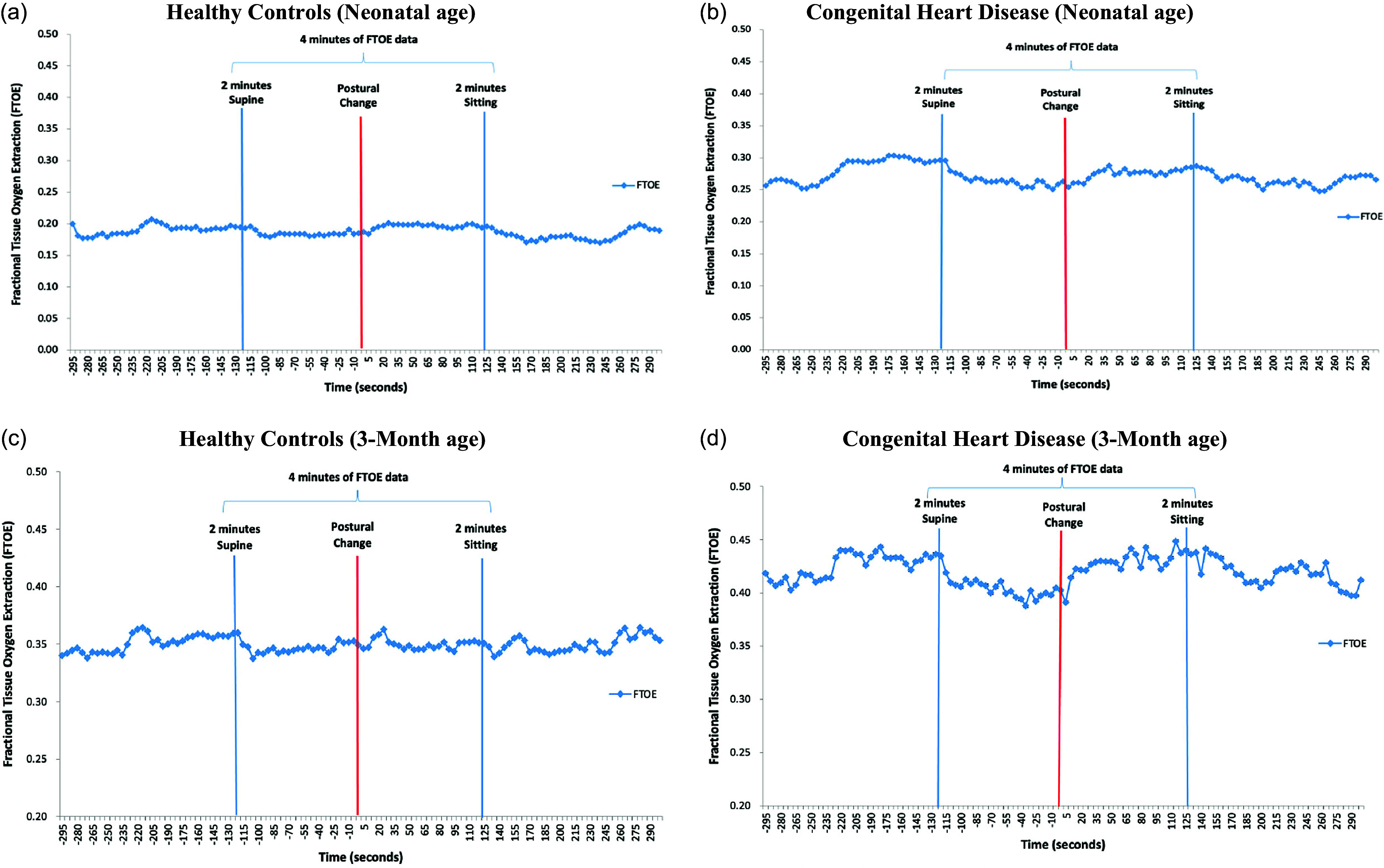
We display a time series of all FTOE values averaged at each time point across all infants in each group at the neonatal (a–b) and the 3-month ages (c–d) for both groups. (a) Healthy controls (Neonatal age). (b) Congenital heart disease (Neonatal age). (c) Healthy controls (3–month age). (d) Congenital heart disease (3-month age).

The flow diagram (Supplemental Fig. 1) depicts the final sample at the neonatal (101 infants: 52 CHD and 49 HC) and 3-month age (108 infants: 45 CHD and 63 HC). 30 CHD infants and 42 HC had data at both ages for the sensitivity analyses. Table 1 displays demographic characteristics of both groups. The CHD group had a variety of heart defects (Table 2). Figures 1 and 2 show a time series of all rcSO_2_ and FTOE.

**Table 1.** Demographics and physiologic measures of the CHD and HC infants at the neonatal and 3-month ages

	HC			CHD			
	*n* (%)	± SD	Mean (%)	*n* (%)	± SD	Mean (%)	*p*-value
**Neonatal age**							
*N*	**49**			**52**			
Bayley Age (months)	49 (100%)	1.31	40.3	52 (100%)	1.08	39.2	**
Mean rcSO_2_ (%)	49 (100%)	6.15	79	52 (100%)	9.21	67	**
Mean SpO_2_ (%)	48 (98.0%)	1.63	97	51 (98.1%)	6.40	92	**
Mean FTOE	49 (100%)	0.07	0.188	50 (96.2%)	0.08	0.271	**
Sex							0.21
Male	26 (53.1%)			21 (40.4%)			
Female	23 (46.9%)			31 (59.6%)			
Race/Ethnicity							0.18
Caucasian	4 (8.2%)			7 (13.5%)			
Latino	30 (61.2%)			36 (69.2%)			
Asian/Other	15 (30.6%)			9 (17.3%)			
**3-Month age**							
*N*	63			45			
Bayley Age (months)	63 (100%)	0.4	2.6	45 (100%)	0.43	2.65	0.73
Mean rcSO_2_ (%)	63 (100%)	6.5	64	45 (100%)	10.0	51	**
Mean SpO_2_ (%)	63 (100%)	1.6	98	45 (100%)	9.8	89	**
Mean FTOE	63 (100%)	0.07	0.351	45 (100%)	0.10	0.422	**
Sex							0.63
Male	31 (49.2%)			20 (44.4%)			
Female	32 (50.8%)			25 (55.6%)			
Race/Ethnicity							0.19
Caucasian	7 (11.1%)			7 (15.6%)			
Latino	38 (60.3%)			28 (62.2)			
Asian/Other	18 (28.5%)			10 (22.2)			

Group comparisons employed either two-sample t-tests or chi-square tests. P-values were 2-sided. CHD = congenital heart disease; FTOE = fractional tissue oxygen extraction; HC = healthy controls; rcSO_2_ = cerebrovascular oxygenation; SpO_2_ = preductal systemic oxygenation; *p < 0.05, **p ≤ 0.001.

**Table 2.** Types of cardiac defects in the CHD group at both ages

	Neonatal *N* = 52 *n* (%)	3-Month *N* = 45 *n* (%)
**Biventricular Cardiac Defects**		
Aortic Stenosis	1 (1.9%)	0
Arch Anomaly + Septal Defect	8 (15.4%)	5 (11.1%)^b^
AVSD with Common Atrium	1 (1.9%)^a^	1 (2.2%)^a^
DORV, VSD type	1 (1.9%)	0
D-TGA and variants	4 (7.7%)^a^	5 (11.1%)
Isolated Arch Anomaly	5 (9.6%)	4 (8.9%)
Isolated Pulmonary Valve Insufficiency	0	1 (2.2%)
L-TGA (with Septal Defect or Coarctation)	2 (3.8%)	1 (2.2%)
Total Anomalous Pulmonary Venous Return	3 (5.8%)^a^	2 (4.4%)
Tetralogy of Fallot	6 (11.5%)^a^	3 (6.7%)^a^
Truncus Arteriosus	3 (5.8%)^a^	2 (4.4%)
**Single Ventricle Cardiac Defects**		
Ebstein’s Anomaly (severe)	0	1 (2.2%)^a^
Hypoplastic Left Heart Syndrome	5 (9.6%)^a^	8 (17.8%)^a^
Single Ventricle with Pulmonary Stenosis/Atresia	9 (17.3%)^a^	9 (20.0%)^a^
Other Single Ventricle	4 (7.7%)^a^	3 (6.7%)^a^

AVSD = atrioventricular septal defect; CHD = congenital heart disease; D-TGA = Dextro Transposition of the Great Arteries; DORV = Double outlet right ventricle; L-TGA = Levo Transposition of the Great Arteries; VSD = ventricular septal defect.

a = Cardiac defects with cyanosis at this timepoint.

b = One subject in this group was cyanotic at this age due to pulmonary artery banding.

### Correlations of rcSO_2_, FTOE, and SpO_2_

rcSO_2_ and FTOE demonstrated a strong inverse correlation across all participants at both ages (*neonatal: r* = –0.92 *p* < 0.001; 3-month: *r* = –0.88, *p* < 0.001). *CHD*: We observed a significant inverse correlation between rcSO_2_ and FTOE at both ages (*neonatal: r* = –0.85, *p* < 0.001; 3-month: *r* = –0.80, *p* < 0.001). *HC*: Similarly, rcSO_2_ and FTOE had a significant inverse correlation between at both ages (*neonatal*: *r* = –0.98, *p* < 0.001; *3-month*: *r* = –0.99, *p* < 0.001).

rcSO_2_ and SpO_2_ were moderately correlated in the sample at both ages (*neonatal*: *r* = 0.64, *p* < 0.001; *3-month*: *r* = 0.53, *p* < 0.001). *CHD*: We found similar results at the neonatal age (*r* = 0.64, *p* < 0.001). However, this correlation became weaker at 3-months (*r* = 0.38, *p* = 0.010). *HC*: We found an inverse correlation, but not significant, at both ages (*neonatal*: *r* = -0.201, *p* = 0.17; *3-month*: *r* = −0.12, *p* = 0.346).

### rcSO_2_ and FTOE between groups

#### CHD versus HC

**Figure 3.**
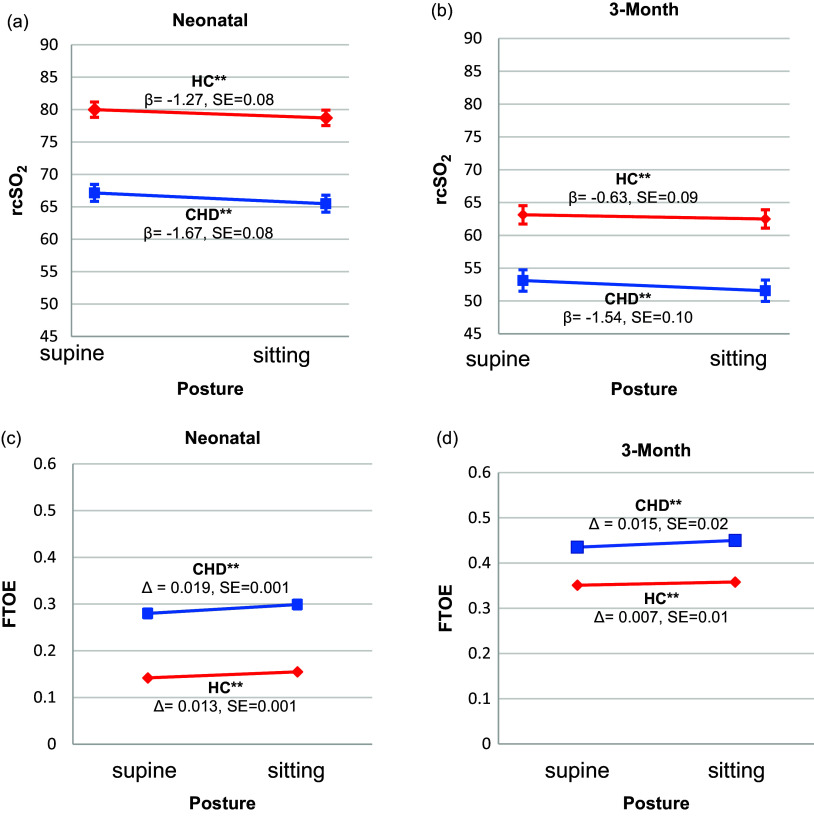
Tilt effects for rcSO_2_ (a–b) and FTOE (c–d) between the groups at the neonatal and 3-month ages. These figures demonstrate the direction of effects for cerebrovascular stability and FTOE response in each group at both the neonatal and 3-month ages. rcSO_2_ and FTOE values are the least square marginal means estimated from linear mixed models for repeated measures that tested the main effects for group and posture and the group-by-posture interaction on rcSO_2_ and FTOE when covarying for postconceptional age (only at the neonatal age), sex, ethnicity, and SpO_2_. Group and group-by-posture interaction effects were significant at both ages for rcSO_2_ and FTOE (p’s<0.001). rcSO_2_ declined from the supine to sitting posture in both groups, but the magnitude of the decline was greater in the CHD group. The red lines for rcSO_2_ represent the HC response after the tilt (*neonatal*: *β* = –1.27, 95% CI [–1.43, –1.11] and the *3-month*: *β* = –0.63, 95% CI [–0.80, –0.46]) (a–b). The blue lines represent the CHD response after the tilt (*neonatal*: *β* = –1.67, 95% CI [–1.83, –1.51] and the *3-month*: *β* = –1.5435, 95% CI [–1.74, –1.33]) (a–b). FTOE values increased from the sitting to supine posture in both groups, but the magnitude of the increase was greater in the CHD group. The red lines for FTOE represent the HC response after the tilt (*neonatal*: *β* = 0.013, 95% CI [0.011, 0.014] and the *3-month*: *β* = 0.007, 95% CI [0.004, 0.009]) (c–d). The blue lines represent the CHD response after the tilt (*neonatal*: *β* = 0.019, 95% CI [0.017, 0.021] and the *3-month*: *β* = 0.015, 95% CI: [0.01, 0.02]) (c–d). Error bars represent standard error. CHD = congenital heart disease; FTOE = fractional tissue oxygen extraction; rcSO_2_ = regional cerebral oxygenation; SpO_2_ = preductal systemic oxygenation. **p ≤ 0.001.

The main effect of group on rcSO_2_ was significant at both ages (Type III Sums of Squares [T3SS]; *neonatal*: *F* = 61.33, df = 92.13, *p* < 0.001; *3-month*: *F* = 46.40, df = 105.20, *p* < 0.001). CHD infants had lower rcSO_2_ (*neonatal*: ∼13%; *3-month*: ∼11%) than HCs in both postures (Figure 3), and disproportionate to the levels predicted by their lower SpO_2_.

Likewise, the main effect of group on FTOE was significant at both ages (T3SS; *neonatal*: *F* = 56.53, df = 92.10, *p* < 0.001; *3-month*: *F* = 18.48, df = 63.99, *p* < 0.001). CHD infants had higher FTOE (*neonatal*: ∼0.14 points; *3-month*: ∼0.09 points) compared to HCs in both postures (Figure 3).

#### Sensitivity analyses

Results were consistent when removing SpO_2_ as a covariate (T3SS; *neonatal*: *F* = 59.89, df = 94.01, *p* < 0.001; *3-month*: *F* = 61.18; df = 103.96, *p* < 0.001). Additionally, the infants with data at both ages yielded similar findings for main effect of group (T3SS; *neonatal*: *F* = 63.72, df = 90.04, *p* < 0.001; *3-month*: *F* = 45.32; df = 105.47, *p* < 0.001). Lastly, the main effect of group was consistent when including postconceptional age as a covariate at 3-months (T3SS; *3-month*: *F* = 35.02; df = 70.03, *p* < 0.001).

### Heart defect subgroups

#### SV versus BV CHD

The main effect of rcSO_2_ was not significant at the neonatal age, but was at 3-months (T3SS; *neonatal*: *F* = 3.44, df = 44.85, *p* = 0.07; *3-month*: *F* = 3.91, df = 41.86, *p* = 0.049). Similarly, the main effect of ventricular type on FTOE trended towards significance at the neonatal age, but was significant at 3-months (T3SS; *neonatal*: *F* = 3.74, df = 44.83, *p* = 0.06; *3-month*: *F* = 4.43, df = 42.08, *p* = 0.04).

#### Cyanotic versus acyanotic CHD

The main effects of cyanosis on rcSO_2_ and FTOE, was significant at the neonatal age (T3SS; *rcSO*_*2*_: *F* = 15.10, df = 45.17, *p* < 0.001; *FTOE*: *F* = 15.47, df = 45.16, *p* < 0.001). However, this was not significant at 3-months (*rcSO*_*2*_: *F* = 1.29, df = 42.01, *p* = 0.26; *FTOE*: *F* = 2.16, df = 42.22, *p* = 0.15).

### Cerebrovascular stability and FTOE response to tilt between groups

#### CHD vs HC

We observed a significant group-by-posture interaction on cerebrovascular stability at both ages (*neonatal*: *β* = –0.39, 95% CI: [–0.63, –0.17], *p* < 0.001; *3-month*: *β* = –0.90; 95% CI: [–1.17, –0.64], *p* < 0.001) indicating lower rcSO_2_ in CHD versus HCs after tilting (Supplemental Table 1). CHD infants showed a greater decline in rcSO_2_ at both ages (*neonatal: β* = –1.67, 95% CI: [–1.83, –1.51], *p* < 0.001; *3-month*: *β* = –1.54, 95% CI: [–1.74, –1.33], *p* < 0.001), than the HC (*neonatal*: *β* = –1.27, 95% CI: [–1.43, –1.11], *p* < 0.001; *3-month*: *β* = –0.63, 95% CI: [–0.80, –0.46], *p* < 0.001) (Figure 3).

We also observed a significant group-by-posture interaction on FTOE at both ages (*neonatal*: *β* = 0.006, 95% CI: [0.003, 0.009], *p* < 0.001; *3-month*: *β* = 0.008; 95% CI: [0.004, 0.012], *p* < 0.001), indicating higher FTOE in CHD compared to HC after tilting (Supplemental Table 2). CHD group had increased FTOE (*neonatal*: *β* = 0.019, 95% CI [0.017, 0.021], *p* < 0.001; *3-month*: *β* = 0.015, 95% CI: [0.012, 0.018], *p* < 0.001) than the HCs (neonatal: *β* = 0.013, 95% CI: [0.011, 0.014], *p* < 0.001; *3-month*: *β* = 0.007, 95% CI: [0.004, 0.009], *p* < 0.001) at both ages (Figure 3).

#### Sensitivity analyses

Estimates for the group-by-posture interaction on rcSO_2_ remained consistent when excluding SpO_2_ from the models (*neonatal*: *β* = –0.10, 95% CI: [–0.08, –0.13] *p* < 0.001; *3-month*: *β* = –0.21, 95% CI: [–0.19, –0.24] *p* < 0.001). Moreover, the mixed-effects models using only infants with data at both ages, revealed comparable results (*neonatal*: *β* = –0.79, 95% CI: [–1.07, –0.51], *p* < 0.001; *3-month*: *β* = –0.75; 95% CI: [–1.09, –0.41], *p* < 0.001).

### Heart defect subgroups

#### SV versus BV CHD

**Figure 4.**
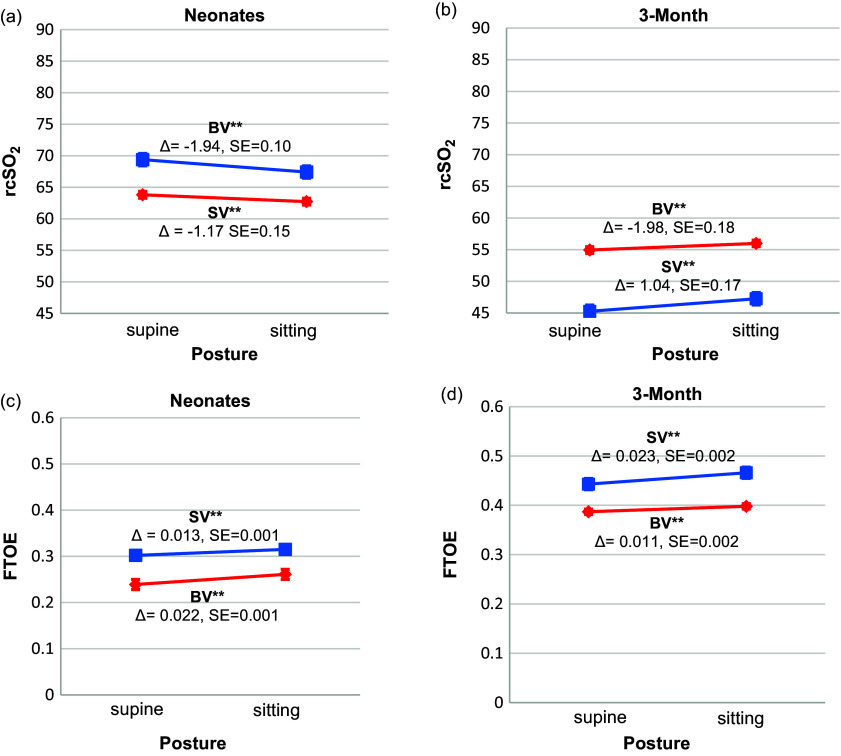
Tilt effects for rcSO_2_ (a–b) and FTOE (c–d) between the biventricular and single ventricle CHD at the neonatal and 3-month ages. These figures demonstrate the direction of effects for the cerebrovascular stability and FTOE between the biventricular (BV) versus single ventricle (SV) CHD groups at both the neonatal and 3-month ages. rcSO_2_ and FTOE values are the least square marginal means estimated from linear mixed models for repeated measures that tested the ventricle type-by-posture interaction on rcSO_2_ and FTOE when covarying for postconceptional age (only at the neonatal age), sex, ethnicity, and SpO_2_. Ventricle type-by-posture interaction effects were significant at both ages for rcSO_2_ and FTOE (p’s<0.001). rcSO_2_ declined from the supine to sitting posture in both groups, but the magnitude of the decline was greater in the BV group at the neonatal age. Conversely, the SV group exhibited a greater decline in rcSO_2_ compared to the BV infants at the 3-month age. The red lines for rcSO_2_ represent the BV CHD response after the tilt (*neonatal: β* = –1.94, 95% CI [–2.15, –1.74] and the *3-month: β* = –1.19, 95% CI [–1.51, –0.88]). The blue lines represent the SV CHD response after the tilt (*neonatal: β* = –1.17, 95% CI [–1.46, –0.87] and the *3-month: β* = –2.43, 95% CI [–2.76, –2.10]) (a–b). FTOE increased from the sitting to supine posture in both groups, but the magnitude of the increase was greater in the BV group at the neonatal age and in the SV group at the 3-month age (a–b). For FTOE, the red lines represent the BV CHD response after the tilt (*neonatal: β* = 0.022, 95% CI [–0.024, –0.020] and the *3-month: β* = 0.011, 95% CI [–0.015, –0.007]) (c–d). The blue lines represent the SV CHD response after the tilt (*neonatal: β* = 0.013, 95% CI [–0.016, –0.009] and the *3-month: β* = 0.023, 95% CI [–0.027, –0.018]) (c–d). The error bars represent standard error. CHD = congenital heart disease; FTOE = fractional tissue oxygen extraction; rcSO_2_ = regional cerebral oxygenation; SpO_2_ = preductal systemic oxygenation. **p ≤ 0.001.

The estimated ventricle type-by-posture interaction on rcSO_2_ was significant at both ages (*neonatal*: *β* = –0.78, 95% CI: [0.42, 1.14], *p* < 0.001; *3-month*: *β* = –0.77; 95% CI: [–1.24, –0.29], *p* = 0.001) (Supplemental Table 3). Both SV and BV CHD had declines in rcSO_2_ after tilts at both ages (Figure 4). However, neonates with BV CHD (*β* = –1.94, 95% CI: [–2.15, –1.74], *p* < 0.001) had a larger decline in rcSO_2_ after tilting compared with the SV group (*β* = –1.17, 95% CI [–1.46, –0.87], *p* < 0.001). Conversely, at the 3-month age, the SV (*β* = –2.43, 95% CI: [–2.76, –2.10], *p* < 0.001) exhibited a larger drop in rcSO_2_ compared to the BV CHD (*β* = –1.19, 95% CI: [–1.51, –0.88], *p* < 0.001). The interaction effect on FTOE was also significant at both ages (*neonatal*: *β* = –0.009, 95% CI: [–0.013, –0.005], *p* = 0.001; *3-month*: *β* = 0.012, 95% CI: [0.006, 0.017], *p* < 0.001) (Supplemental Table 4). Both SV and BV infants experienced an increase in FTOE after tilting at both ages (Figure 4). This increase was greater in BV infants at the neonatal age (*BV*: *β* = 0.022, 95% CI: [–0.024, –0.020], *p* = 0.001; *SV*: *β* = 0.013, 95% CI: [–0.016, –0.009], *p* = 0.002). However, it did not persist at 3-months, where SV infants had increased FTOE (*BV*: *β* = 0.011, 95% CI: [–0.015, –0.007], *p* = 0.002; *SV*: *β* = 0.023, 95% CI: [–0.027, –0.018], *p* = 0.002).

#### Cyanotic versus acyanotic CHD

**Figure 5.**
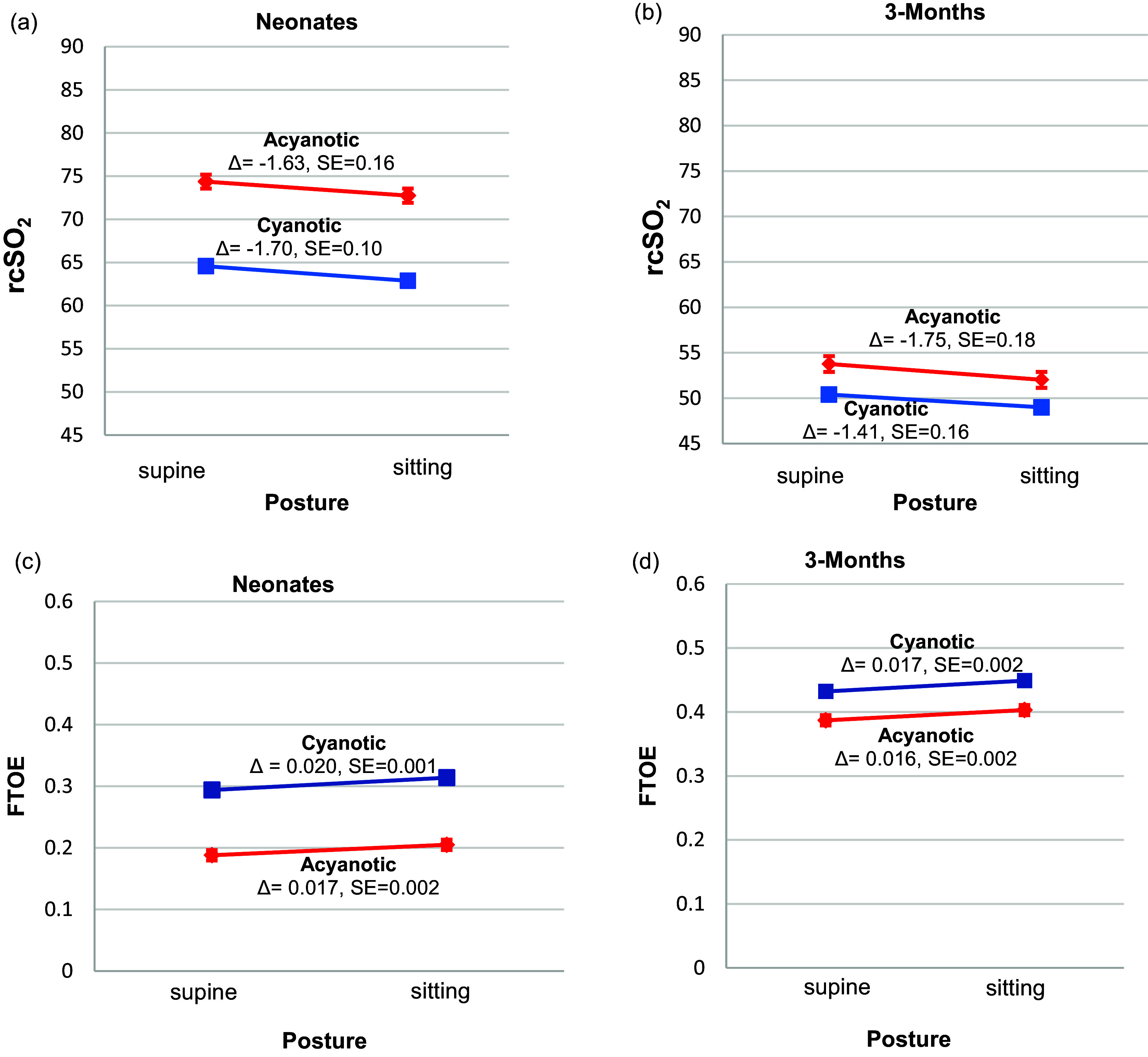
Tilt effects for rcSO_2_ (a–b) and FTOE (c–d) between the cyanotic and acyanotic CHD at the neonatal and 3-month ages. These figures demonstrate the direction of effects for the cerebrovascular stability between the cyanotic versus acyanotic CHD groups at the neonatal and 3-month age. rcSO_2_ and FTOE values are the least square marginal means estimated from linear mixed models for repeated measures that tested the cyanosis-by-posture interaction on rcSO_2_ and FTOE when covarying for postconceptional age (only at the neonatal age), sex, ethnicity, and SpO_2_. Both cyanotic and acyanotic infants with CHD experienced a decline in rcSO_2_ during postural changes from supine to sitting at both ages, although cyanotic infants showed a greater decline at the neonatal age and acyanotic infants exhibited a greater decline at the 3-month age. The red lines for rcSO_2_ represent the acyanotic CHD response after the tilt (*neonatal: β* = –1.63, 95% CI [–1.94, –1.31] and the *3-month: β* = –1.75, 95% CI [–2.11, –1.39]) (a–b). The blue lines represent the cyanotic CHD response after the tilt (*neonatal: β* = –1.70, 95% CI [–1.89, –1.49] and the *3-month: β* = –1.41, 95% CI [–1.72, –1.09]) (a–b). FTOE increased from the sitting to supine posture in both groups, but the magnitude of the increase was greater in the cyanotic group at both ages. The red lines for FTOE represent the acyanotic CHD response after the tilt (*neonatal: β* = 0.017, 95% CI [0.013, 0.020] and the *3-month: β* = 0.016, 95% CI [0.011, 0.020]) (c–d). The blue lines represent the cyanotic CHD response after the tilt (*neonatal: β* = 0.020, 95% CI [0.018, 0.022] and the *3-month: β* = 0.017, 95% CI [–0.013, –0.021]) (c–d). Error bars represent standard error. CHD = congenital heart disease; FTOE = fractional tissue oxygen extraction; rcSO_2_ = regional cerebral oxygenation; SpO_2_ = preductal systemic oxygenation.

The cyanosis-by-posture interaction on rcSO_2_ was not significant at either age (*neonatal*: *β* = 0.07, 95% CI: [–0.44, 0.30], *p* = 0.72; *3-month*: *β* = –0.34; 95% CI: [–0.14, 0.82], *p* = 0.16) (Supplemental Table 5). However, the patterns of the decline differed, cyanotic infants had a greater decline at the neonatal age and acyanotic infants exhibited a greater decline at the 3-month age (Figure 5). Similarly, the cyanosis-by-posture interaction effect on FTOE was not significant at both ages (*neonatal*: *β* = 0.003, 95% CI: [–0.001, 0.007], *p* = 0.121; *3-month*: *β* = 0.001; 95% CI: [–0.004, 0.007], *p* = 0.62 (Supplemental Table 6). Both groups increased FTOE after tilts at both ages (Figure 5). The magnitude of increase in FTOE was greater in cyanotic CHD at the neonatal age (*acyanotic*: *β* = 0.017, 95% CI: [0.01 0.02], *p* < 0.001; *cyanotic*: *β* = 0.02, 95% CI: [0.018, 0.022], *p* < 0.001) and at the 3-month age (*acyanotic*: *β* = 0.02, 95% CI: [0.011, 0.020], *p* = 0.002; *cyanotic*: *β* = 0.017, 95% CI: [0.013, 0.021], *p* = 0.002).

### Exploratory analysis: systemic hypoxemia

We found significant main effects for group at both ages (T3SS; *neonatal*: *F* = 29.44, df = 92.94, *p* < 0.001; *3-month*: *F* = 55.50, df = 104, *p* < 0.001) and for the group-by-posture interaction at the neonatal (*β* = –0.19, 95% CI: [–0.35, –0.04], *p* = 0.01), but not at the 3-month age (*β* = 0.12, 95% CI: [–0.05, 0.30], *p* = 0.18). Thus, group differences in the effects of posture on rcSO_2_ at 3-months were specific to the brain and did not mirror the effects of SpO_2_. The CHD group revealed significant effects of posture on SpO_2_ at 3-months (*neonatal*: *β* = –0.13, 95% CI: [–0.01, 0.27], *p* = 0.07; *3-month*: *β* = 0.18, 95% CI: [0.34, 0.03], *p* = 0.01).

## Discussion

### Cerebral oxygenation and FTOE differences between groups

#### CHD versus HC

rcSO_2_ values (∼60%) were 11–13 points lower, on average, in the CHD group at both ages compared to HC, consistent with previous findings [14]. This reduction likely results from oxygenated and deoxygenated blood mixing due to heart defects. Our CHD group exhibited a moderate to weak correlation between rcSO_2_ and SpO_2_, suggesting that lower SpO_2_ may contribute to rcSO_2_ reductions, potentially failing to meet cerebral metabolic needs (particularly during times of increased demand or stress) and increasing the risk of hypoxic injury [9,14]. In our cohort, this effect persisted to the 3-month age.

rcSO_2_ and FTOE were inversely correlated in both groups at both ages. This correlation was stronger in HC, indicating their greater regulation between rcSO_2_ and FTOE. Larger rcSO_2_ reductions in CHD infants likely contribute to their higher FTOE, a pattern also seen in preterm infants with intraventricular hemorrhage compared to those without [15].

FTOE was 0.09–0.14 points higher in CHD infants than HCs at both ages, consistent with findings showing higher preoperative FTOE and lower CBF in SV CHD neonates compared to HCs [16]. We theorize that increased FTOE reflects decreased oxygen delivery due to inadequate oxygen supply from heart defects, as maximal extraction is unexpected as a compensatory mechanism during early infancy. Research on FTOE beyond the immediate postoperative period remains limited in CHD infants, however, our neonatal findings align with previous findings demonstrating increased FTOE in this population [17].

Both groups exhibited an expected developmental decline in rcSO_2_ and increased FTOE across ages. These changes resulted in group differences in oxygenation and extraction that were consistent in magnitude across both ages, which suggests persistent cerebral hypoxia in CHD infants during the first 3-months of life (despite 87% having undergone corrective or palliative surgery). Specifically, rcSO_2_ in CHD infants dropped from 67% neonatally to 51% at 3-months, while FTOE increased from 0.271 to 0.422. Average neonatal FTOE (0.271) is lower than in healthy adults as reported in studies (0.328 to 0.380) [18,19]. The age-related increase in FTOE likely reflects the natural decline in fetal hemoglobin, which has higher oxygen affinity than adult hemoglobin, bringing values closer to those of a healthy child or adult [20].

However, the mechanisms driving persistent cerebral hypoxia post-surgery, particularly in BV CHD, remain unclear, as only 21% of the BV group remained cyanotic at the 3-month age. One likely driver is the transition from fetal to adult hemoglobin, beginning in late gestation and typically complete by 3-months or after blood transfusions [21]. A previous study on preterm infants found that lower hemoglobin correlated with decreased rcSO_2_ and increased FTOE due to reduced oxygen transport [22]. Adult hemoglobin facilitates oxygen unloading, lowering cerebral oxygenation [23]. Thus, rcSO_2_ reductions in healthy infants may reflect normal physiological adaptation via lower CBF, however, CHD infants had reduced cerebral oxygenation and compromised cerebral hemodynamics [6], suggesting their lower rcSO_2_ may indicate physiological stress. Physiologic anemia also accompanies this hemoglobin transition, with hematocrit levels lowest around 6 weeks. rcSO_2_ often tracks hematocrit levels [24], which typically increases and stabilizes by 3-months of age [25]. Cyanotic CHD infants might demonstrate higher hematocrits than acyanotic, but this modest compensatory polycythemia often manifests later in life. Our study did not collect hemoglobin nor hematocrit, limiting insight into the relationship between hemoglobin, rcSO_2_, and FTOE. Notably, our primary outcomes were preserved when controlling for oxygen saturation, a known driver for hematocrit differences. Future studies should include hemoglobin measurements to further explore the mechanisms of persistent cerebral hypoxia in CHD.

### Heart defect subgroups

#### SV versus BV CHD

SV rcSO_2_ levels were 4.96% to 6.17% lower than BV at both ages, with significant differences only at the 3-month age, suggesting a progressive hemodynamic divergence. SV physiology relies on one ventricle to supply both systemic and pulmonary blood flow, causing volume overload and arterial desaturation due to mixing of oxygenated and deoxygenated blood [26]. Significant rcSO_2_ reductions at 3-months suggests that cumulative effects may further reduce rcSO_2_ in SV compared to BV CHD. Limited pediatric studies compare rcSO_2_ between ventricle type defects, but one adult study found persistently reduced rcSO_2_ and higher hemoglobin in SV versus BV CHD [27]. Other reports demonstrated preoperative cerebral hypoperfusion and postoperative rcSO_2_ declines in CHD infants with SV defects [8], implying that SV physiology imposes a lasting, greater hemodynamic burden, reducing rcSO_2_ into adulthood.

FTOE in SV CHD was 0.054–0.068 points higher than BV at both ages, with significance only at 3-months. This aligns with another study finding lower rcSO_2_ and higher FTOE in SV versus BV CHD infants before and after blood transfusions [28]. Likewise, a report found increased FTOE in older infants with SV versus BV CHD pre- and postoperatively [29]. These findings corroborate our results of lower rcSO_2_ in BV neonates, indicating that increased FTOE compensates for lower rcSO_2_ in BV neonates, but this shifts by 3-months, likely due to growing hemodynamic strains from SV defects and incomplete cerebral recovery or adaptation after surgery [16].

#### Cyanotic versus acyanotic CHD

Neonates with cyanotic defects had ∼ 10% lower rcSO_2_ than acyanotic, with differences persisting at 3-months but attenuating to ∼ 3% lower, perhaps due to age and intervening surgery. We anticipated this finding because cerebral oxygenation is a function of systemic saturation, which naturally results in lower rcSO_2_ in cyanotic infants. Thus, observed rcSO_2_ differences between SV and BV groups are unlikely due to cyanosis alone.

Studies comparing rcSO_2_ between cyanotic heart lesions remains scarce. One contrasted our neonatal findings, found no significant differences in rcSO_2_ between lesion groups, possibly due to a smaller sample size [30]. However, prior studies indicated lower CBF via brain MRI in the thalamus, basal ganglia, and occipital white matter in cyanotic neonates, which supports a mechanism for lower brain oxygenation [31]. Results at 3-months did not reach significance, however, findings suggests cyanotic CHD infants’ potential reliance on FTOE to meet cerebral metabolic demands. Variability of cyanotic heart defects and surgical effects may contribute to these observations. Cyanotic defects have both increased and decreased pulmonary blood flow [32,33], resulting in differing levels of cerebral oxygen delivery at birth. Early clinical interventions may help stabilize rcSO_2_ and minimize the effects of these variations in oxygen delivery by 3-months.

Cyanotic CHD also trended towards increased FTOE than acyanotic at both ages. Our findings aligned with studies in other populations showing increased FTOE as oxygen saturation (e.g., preterm infants [34] and adults) [35]. Likewise, another study revealed that acyanotic CHD infants exhibited significantly higher FTOE compared to cyanotic [36]. Differences in findings may result from differing measurement techniques, like the FORE-SIGHT monitor, and sample size variations.

### Cerebrovascular stability differences and FTOE response to tilt between groups

#### CHD versus HC

Our findings suggest reduced cerebrovascular stability in CHD infants preoperatively, persisting to 3-months post-surgery. rcSO_2_ declined while FTOE increased significantly after tilting to a seated position at both ages compared to healthy infants.

The reduced cerebrovascular stability in CHD infants align with our prior work, which showed a significant decline in rcSO_2_ after tilting in CHD neonates [9]. Reports measuring cerebrovascular stability in CHD infants are limited, however, existing studies in preterm infants have yielded inconsistent rcSO_2_ responses to position changes [37]. Some reported significant declines after tilting [38,39], whereas others found no differences [40,41], possibly due to variations in preterm study populations [40,41], tilt angles employed, rest durations between position changes [42], or time durations within each posture [42,43]. We observed significant rcSO_2_ decreases after tilting between the CHD and HC groups (0.5% at the neonatal age and ∼ 0.9% at 3-months), however, their clinical relevance remains uncertain when considered independently. Nevertheless, the persistence to 3-months suggests continued physiological differences between the two groups, despite 42% of the CHD cohort having undergone corrective cardiac surgery prior to that timepoint. These findings suggest enduring cerebrovascular vulnerabilities in high-risk infants, like those with CHD.

Perfusion studies via MRI or cranial ultrasound, for example, have reported reduced oxygen delivery [44], and reduced CBF [45] in preoperative CHD infants compared with HC. Similarly, a postmortem study of brains of children with CHD showed diffuse gliosis, suggesting chronic cerebral hypoperfusion may persist from infancy into adulthood [46]. These studies, despite using different methodologies from ours, support the changes in CBF and oxygenation in CHD infants and provide insight into the mechanism for reduced cerebrovascular stability.

Likewise, increased FTOE after tilting in the CHD group align with our previous work in a smaller CHD sample [17]. The magnitude of FTOE increase after tilting was also greater at both ages for both groups, suggesting higher metabolic needs and difficulties sustaining sufficient cerebral perfusion during activity [47]. In previous studies, CHD infants exhibited up to 35% greater total energy expenditure compared to healthy age-matched controls [48]. This increased oxygen consumption [49], in addition to impaired CA, can overwhelm the infant’s ability to sustain sufficient cerebral perfusion [50], leading to increased FTOE as the body attempts to meet the elevated metabolic demands [17]. This aligns with another study that found associations between increased FTOE and impaired CA in CHD neonates [6]. CA depends on the cerebral blood vessels’ ability to dilate and constrict based on the changes in perfusion pressure, and in the setting of maximal FTOE, therefore may not be ability to further vasodilate and allow for autoregulation [51].

### Heart defect subgroups

#### SV versus BV CHD

We observed significantly reduced cerebrovascular stability for BV at the neonatal age and for SV defects at 3-months. This contrasted our previous study, where rcSO_2_ did not differ by ventricle type in CHD neonates [9]. Differences in findings may result from our larger sample size, which included 52 CHD neonates compared to 28, previously. The pattern reversed at the 3-months, with reduced cerebrovascular stability and increased FTOE in SV relative to BV CHD (full cardiac repair in 79% prior to 3-months). SV defects are more severe, so poorer cerebrovascular response were expected. Reduced cerebrovascular stability in BV CHD cannot be explained by oxygen saturation (50% of BV neonates were cyanotic and 100% of SV were cyanotic) nor by differences in cardiac output at the neonatal age. At 3-months, most of the BV defects had completely repaired heart lesions compared to the SV whose defects were palliated. Our findings suggest that reparative surgery may have a beneficial effect toward cerebrovascular stability.

The FTOE response demonstrated similar findings, where BV defects had increased FTOE after tilting compared to SV at the neonatal age, while SV defects exhibited a greater increase at the 3-month age. Studies on FTOE during postural changes in SV CHD infants remain limited, however, one report demonstrated increased FTOE in neonates with SV CHD during an active state compared to a sedated state postoperatively [16]. Studies in preterm infants further support our findings, revealing similar increases in FTOE after changes in positions [34]. Thus, findings suggest that periods of activity, such as during a postural tilt, may increase FTOE to satisfy increased metabolic demands. Again, our 3-month findings indicate that repairing the defect may benefit this response. These findings also suggest the presence of an underlying abnormality distinct from SpO_2_ alone. Infants with BV hearts may have a greater capacity to augment cardiac output or extract oxygen more effectively during periods of metabolic demand, which would explain the observed shift at the 3-month age.

#### Cyanotic versus acyanotic CHD

Cyanotic CHD did not exhibit reduced cerebrovascular stability compared to acyanotic CHD at either age. This suggests that SpO_2_ from intracardiac mixing does not alone account for the abnormalities we observed in our CHD cohort. At the neonatal age, cyanotic defects had significant increases in FTOE after tilting, which contrasted the ventricle type results. We expected reduced cerebrovascular stability in cyanotic CHD, as these infants typically have poorer outcomes. Instead, we observed preserved cerebrovascular stability in cyanotic neonates despite significant increases in FTOE. The explanation remains unclear, and a gap in the literature exists for this topic. However, these results aligned with our prior study showing cerebrovascular stability in neonatal cyanotic CHD [9]. One study suggested that oxygen supply and consumption influences FTOE, as increased FTOE may result from decreased CBF [52], which could explain the increases in FTOE in cyanotic infants compared to acyanotic infants after tilting at the neonatal age. We have not found literature on the relationship between FTOE and tilts in cyanotic CHD infants. However, reports in preterm populations show increased FTOE with reductions in rcSO_2_ during position changes [34]. Thus, acutely ill infants experience difficulty maintaining adequate rcSO_2_, leading to increased FTOE, as the body attempts to meet heightened metabolic demands.

### Probable consequences of poorer cerebrovascular health

Cerebral hypoxia, reduced cerebrovascular stability, and increased FTOE implies that CHD infants may experience inadequate cerebral perfusion, especially during periods of physiological stress. Lower rcSO_2_ may therefore contribute to the poorer long-term developmental outcomes in CHD infants compared to HCs, even after surgical repair of their heart defects [53]. CHD newborns have significant associations of reduced oxygen delivery with lower cortical gray matter volumes and less gyrification on brain MRIs [44], potentially due to chronic hypoperfusion. Other findings showed that fluctuations in CBF during the intra- and postoperative periods associated with brain injury and lower neurodevelopmental scores at 1-year old in CHD infants [54,55]. Increased FTOE after tilting may be a compensatory response to meet cerebral metabolic needs in response to the lower rcSO_2_ post-tilt [56 –58], potentially worsening CA [6]. Continuous cerebral hypoperfusion [46], vacillations in flow, and hypoxia likely injure neurons and glial cells. This injury likely contributes to abnormal brain maturity, manifested as reduced gray and white matter and cortical gyrification, and thus neurodevelopmental disabilities (NDDs) [44].

## Limitations

Our study had several limitations. First, cerebrovascular stability, using changes in rcSO_2_ as a proxy for CBF, assumed similar cerebral metabolic rates across groups and postures. We minimized metabolic variability by standardizing infant states during data collection. However, cerebrovascular stability has not been definitively validated in literature, with one study with a small sample size showing a moderate, nonsignificant correlation [17 ]. Further research comparing cerebrovascular stability to standard CA methods is necessary.

Second, blood transfusions may have confounded results in CHD neonates. Transfusions replace fetal hemoglobin with adult hemoglobin, lowering oxygen affinity, reducing CBF, and increasing FTOE. These factors complicate FTOE interpretation in this population and highlight the need to consider fetal-to-adult hemoglobin ratios in future studies using hemoglobin electrophoresis.

Third, the use of a single modality, NIRS, to assess cerebrovascular stability without supporting laboratory hemoglobin data or echocardiographic measures limits the clinical relevance of our findings. Echocardiographic insights such as ventricular function, residual lesions, or shunting would provide context for interpreting cerebral oxygenation patterns. While this study offers preliminary data, future studies should incorporate multi-modal approaches to validate these findings and determine their clinical relevance. Fourth, varying cardiac output and physiology likely influenced CBF and cerebrovascular stability across time, as we assessed infants both before and after palliative and corrective heart surgeries. Our limited sample size prevented analysis of specific CHD lesions. Thus, we combined data across different lesions to provide a broader understanding of rcSO_2_ and FTOE trends in CHD infants, recognizing that lesion-specific variability in clinical presentation may impact outcomes. Medical acuity, hospitalization timing, and impending surgery resulted in missed visits. Nevertheless, our sensitivity analyses of those with consecutive measurements found minimal differences. Variability in CHD defect types may also limit the generalizability of our findings.

## Conclusions

We found evidence of poorer cerebrovascular health (i.e., sustained cerebral hypoxia, reduced cerebrovascular stability, and increased FTOE) in neonates and 3-month infants with CHD compared to HCs. These abnormalities persisted beyond the neonatal period, even after cardiac interventions, suggesting that sustained disturbances in cerebrovascular health may contribute to brain injury and NDDs in CHD infants. Future studies should assess our measures of cerebrovascular health as predictors of NDDs in children with CHD, as they may serve as biomarkers to identify high-risk infants. We could then evaluate interventions to improve cerebrovascular health and assess their potential to prevent or attenuate NDDs in children with CHD. Our technique to measure cerebrovascular health is noninvasive, low-cost, and easily replicable in various settings (e.g., clinic and home) and diverse pediatric populations (e.g., high-risk and healthy), offering a potentially valuable tool for clinicians to closely monitor infants at an increased risk for poorer cerebrovascular health and neurodevelopmental delays.

## Supporting information

10.1017/cts.2025.10106.sm001 Tran et al. supplementary material Tran et al. supplementary material

## Supplementary material

The supplementary material for this article can be found at https://doi.org/10.1017/cts.2025.10106
