# Supplementary material for: Neonatal and 3-month cerebrovascular oxygenation, stability, and extraction in congenital heart disease versus control infants
Source: J Clin Transl Sci. 2025 Jul 21;9(1):e175. doi: 10.1017/cts.2025.10106 (PMC12444710; doi:10.1017/cts.2025.10106)
Supplement: Tran et al. supplementary material [file S2059866125101064sup001.docx]

**Supplemental Figure 1. Flow Diagram of Participants at the Neonatal and 3-month Ages.** **
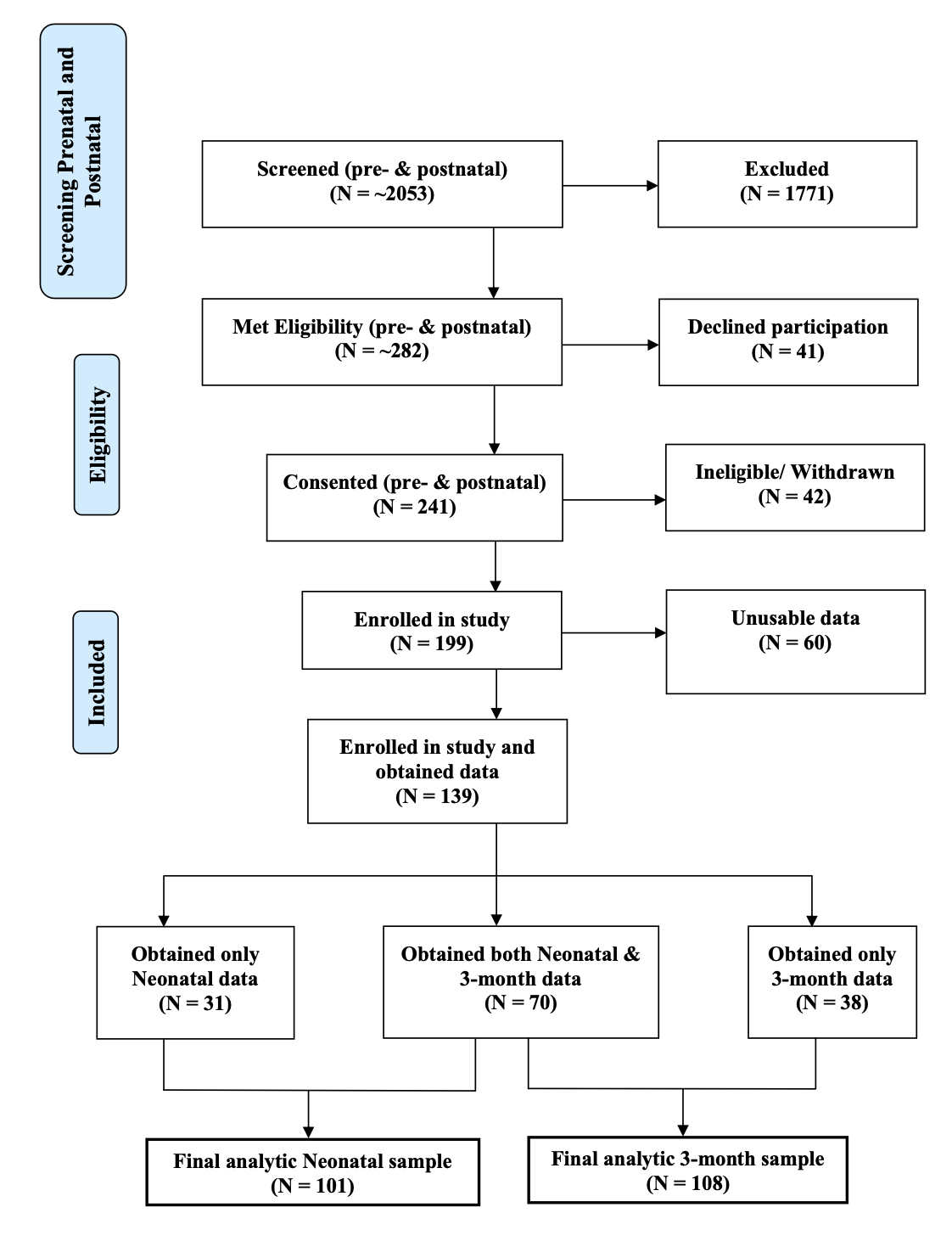
**

**Supplemental Table 1.** **Effects of Group (CHD vs. HC infants) on Cerebrovascular Stability at the Neonatal and 3-month Ages.**

| **Neonatal Age** | | | | | |
| --- | --- | --- | --- | --- | --- |
| **rcSO_2_** | **N=101** | **β** | **Standard Error** | **95% CI** | **p-value** |
| **Postconceptional Age (weeks)** |  | -1.04 | 0.65 | (-2.32,0.24) | 0.11 |
| **Sex** |  | -2.17 | 1.52 | (-5.19,0.86) | 0.15 |
| **Ethnicity** |  | -0.96 | 0.96 | (-2.89,0.95) | 0.32 |
| **SpO_2_ (%)** |  | 0.10 | 0.01 | (0.07,0.12) | ** |
| **Posture** |  |  |  |  |  |
| **Supine** |  | Ref | - | - | - |
| **Sitting** |  | -1.66 | 0.08 | (1.51,1.83) | ** |
| **Group** |  |  |  |  |  |
| **HC** | 49 | Ref | - | - | - |
| **CHD** | 52 | -13.24 | 1.66 | (-9.93,-16.55) | ** |
| **Group-by-Posture** |  | -0.39 | 0.12 | (-0.63,-0.17) | ** |
| **3-Month Age** | | | | | |
| **rcSO_2_** | **N=108** | **β** | **Standard Error** | **95% CI** | **p-value** |
| **Sex** |  | -1.99 | 1.51 | (-4.98,0.10) | 0.19 |
| **Ethnicity** |  | 0.06 | 0.86 | (-1.64,1.76) | 0.94 |
| **SpO_2_ (%)** |  | 0.21 | 0.01 | (0.18,0.23) | ** |
| **Posture** |  |  |  |  |  |
| **Supine** |  | Ref | - | - | - |
| **Sitting** |  | -1.53 | 0.10 | (1.33,1.74) | ** |
| **Group** |  |  |  |  |  |
| **HC** | 63 | Ref | - | - | - |
| **CHD** | 45 | -10.92 | 1.53 | (-7.87,-13.97) | ** |
| **Group-by-Posture** |  | -0.90 | 0.13 | (-1.17,-0.64) | ** |

Mixed effects regression models for repeated measures assessed the associations of group (CHD, HC) and posture with rcSO_2_ values. The main effect of group tested our hypothesis of reduced cerebral oxygen saturation, and the group-by-posture interaction tested our hypothesis of cerebrovascular stability in infants with CHD. Covariates in the model were postconceptional age (only at the neonatal age), SpO_2,_ sex, and ethnicity.

CHD = congenital heart disease; HC = healthy controls; rcSO_2_ = regional cerebral oxygenation; SpO_2_ = preductal systemic oxygenation

*p<0.05, **p≤0.001

**Supplemental Table 2.** **Effects of Group (CHD vs. HC infants) on FTOE at the Neonatal and 3-month Ages.**

| **Neonatal Age** | | | | | |
| --- | --- | --- | --- | --- | --- |
| **FTOE** | **N=99** | **β** | **Standard Error** | **95% CI** | **p-value** |
| **Postconceptional Age (weeks)** |  | 0.013 | 0.01 | (-0.001, 0.03) | 0.07 |
| **Sex** |  | 0.018 | 0.02 | (0.02,0.05) | 0.29 |
| **Ethnicity** |  | -0.058 | 0.03 | (-0.13,0.01) | 0.10 |
| **SpO_2_ (%)** |  | 0.008 | 0.01 | (0.01,0.01) | ** |
| **Posture** |  |  |  |  |  |
| **Supine** |  | -0.016 | 0.001 | (-0.02,-0.017) | ** |
| **Sitting** |  | Ref | - | - | - |
| **Group** |  |  |  |  |  |
| **HC** | 49 | -0.141 | 0.02 | (-0.18, -0.11) | ** |
| **CHD** | 50 | Ref | - | - | - |
| **Group-by-Posture** |  | 0.006 | 0.001 | (0.004, 0.009) | ** |
| **3-Month Age** | | | | | |
| **FTOE** | **N=108** | **β** | **Standard Error** | **95% CI** | **p-value** |
| **Postconceptional Age (weeks)** |  | -0.010 | 0.01 | (-0.02, 0.01) | 0.27 |
| **Sex** |  | 0.003 | 0.02 | (-0.03, 0.04) | 0.87 |
| **Ethnicity** |  | -0.004 | 0.01 | (-0.03, 0.04) | 0.73 |
| **SpO_2_ (%)** |  | 0.004 | 0.0001 | (0.00, 0.00) | ** |
| **Posture** |  |  |  |  |  |
| **Supine** |  | -0.015 | 0.002 | (-0.02, -0.01) | ** |
| **Sitting** |  | Ref | - | - | - |
| **Group** |  |  |  |  |  |
| **HC** | 63 | -0.09 | 0.02 | (-0.14, -0.05) | ** |
| **CHD** | 45 | Ref | - | - | - |
| **Group-by-Posture** |  | 0.008 | 0.002 | (0.004, 0.012] | ** |

Mixed effects regression models for repeated measures assessed the associations of group (CHD, HC) and posture with FTOE values. The main effect of group tested our hypothesis of reduced cerebral oxygen saturation, and the group-by-posture interaction tested our hypothesis of FTOE in infants with CHD. Covariates in the model were postconceptional age (only at the neonatal age), SpO_2,_ sex, and ethnicity.

CHD = congenital heart disease; FTOE = fractional tissue oxygen extraction; HC = healthy controls; SpO_2_ = preductal systemic oxygenation

*p<0.05, **p≤0.001

**Supplemental Table 3. Effects of Single Ventricle vs. Biventricular CHD on Cerebrovascular Stability at the Neonatal and 3-month Ages.**

| **Neonatal Age** | | | | | |
| --- | --- | --- | --- | --- | --- |
| **rcSO_2_** | **N=52** | **β** | **Standard Error** | **95% CI** | **p-value** |
| **Postconceptional Age (weeks)** |  | -1.91 | 1.21 | (-4.35,0.53) | 0.12 |
| **Sex** |  | -2.16 | 2.62 | (-7.43, 3.12) | 0.41 |
| **Ethnicity** |  | -1.45 | 1.96 | (-5.40, 2.49) | 0.46 |
| **SpO_2_ (%)** |  | 0.07 | 0.01 | (0.04,0.10) | ** |
| **Single ventricle** |  |  |  |  |  |
| **No** | 35 | Ref | - | - | - |
| **Yes** | 17 | -4.96 | 2.73 | (0.53, -10.45) | 0.08 |
| **Posture** |  |  |  |  |  |
| **Supine** |  | Ref | - | - | - |
| **Sitting** |  | 1.17 | 0.15 | (0.87, 1.46) | ** |
| **Single ventricle-by-posture** |  | -0.78 | 0.18 | (0.42, 1.14) | ** |
| **3-Month Age** | | | | | |
| **rcSO_2_** | **N=45** | **β** | **Standard Error** | **95% CI** | **p-value** |
| **Sex** |  | -3.86 | 2.65 | (-9.21, 1.49) | 0.15 |
| **Ethnicity** |  | 0.10 | 1.73 | (-3.60, 3.39) | 0.95 |
| **SpO_2_ (%)** |  | 0.28 | 0.02 | (0.24, 0.32) | ** |
| **Single Ventricle** |  |  |  |  |  |
| **No** | 24 | Ref | - | - | - |
| **Yes** | 21 | -5.82 | 2.75 | (0.27, 11.38) | 0.04* |
| **Posture** |  |  |  |  |  |
| **Supine** |  | Ref | - | - | - |
| **Sitting** |  | 1.96 | 0.18 | (1.62, 2.30) | ** |
| **Single ventricle-by-posture** |  | -0.77 | 0.23 | (-1.24, -0.29) | ** |

Mixed-effects regression models for repeated measures assessed the associations of single ventricle vs. biventricular defects and posture with rcSO_2_ values. The main effect of ventricle type tested our hypothesis of reduced cerebral oxygen saturation, and the single ventricle-by-posture interaction tested our hypothesis of decreased cerebrovascular stability in single ventricle CHD. The estimated main effect of ventricular type on rcSO_2_ was not statistically significant at the neonatal age, but was significant at 3-months, while controlling for preductal SpO_2_, ethnicity, sex, and postconceptional age. The estimated ventricle type-by-posture interaction effect on rcSO_2_ values was significant at both ages. Covariates in the model were postconceptional age (only at the neonatal age), SpO_2,_ ethnicity, and sex.

CHD = congenital heart disease; rcSO_2_ = regional cerebral oxygenation; SpO_2_ = preductal systemic oxygenation.
*p<0.05, **p≤0.001

**Supplemental Table 4. Effects of Single Ventricle vs. Biventricular CHD on FTOE at the Neonatal and 3-month Ages.**

| **Neonatal Age** | | | | | |
| --- | --- | --- | --- | --- | --- |
| **FTOE** | **N=52** | **β** | **Standard Error** | **95% CI** | **p-value** |
| **Postconceptional Age (weeks)** |  | 0.021 | 0.013 | (-0.006, 0.049) | 0.117 |
| **Sex** |  | 0.029 | 0.030 | (-0.031, 0.88) | 0.337 |
| **Ethnicity** |  | 0.011 | 0.022 | (-0.034, 0.056) | 0.625 |
| **SpO_2_ (%)** |  | 0.008 | 0.000 | (0.008, 0.008) | ** |
| **Single ventricle** |  |  |  |  |  |
| **Yes** | 18 | Ref | - | - | - |
| **No** | 34 | -0.054 | 0.030 | (-0.115, 0.007) | 0.082 |
| **Posture** |  |  |  |  |  |
| **Supine** |  | -0.013 | 0.002 | (-0.016, -0.009) | ** |
| **Sitting** |  | - | - | - | - |
| **Single ventricle-by-posture** |  | -0.009 | 0.002 | (-0.013, -0.005) | ** |
| **3-Month Age** | | | | | |
| **FTOE** | **N=45** | **β** | **Standard Error** | **95% CI** | **p-value** |
| **Sex** |  | 0.044 | 0.028 | (-0.014, 0.101) | 0.132 |
| **Ethnicity** |  | 0.002 | 0.019 | (-0.035, 0.040) | 0.911 |
| **SpO_2_ (%)** |  | 0.004 | 0.000 | (0.003, 0.004) | ** |
| **Single Ventricle** |  |  |  |  |  |
| **Yes** | 21 | Ref | - | - | - |
| **No** | 24 | -0.068 | 0.030 | (-0.128, -0.008) | 0.027* |
| **Posture** |  |  |  |  |  |
| **Supine** |  | -0.023 | 0.002 | (-0.027, -0.018) | ** |
| **Sitting** |  | Ref | - | - | - |
| **Single ventricle-by-posture** |  | 0.012 | 0.003 | (0.006, 0.017) | ** |

Mixed-effects regression models for repeated measures assessed the associations of single ventricle vs. biventricular defects and posture with FTOE values. The main effect of ventricle type tested our hypothesis of reduced cerebral oxygen saturation, and the single ventricle-by-posture interaction tested our hypothesis of increased FTOE in single ventricle CHD. The estimated main effect of ventricular type on FTOE values showed no statistical significance at the neonatal age but was significant at the 3-month age, while controlling for SpO_2_, ethnicity, sex, and postconceptional age. The estimated ventricle type-by-posture interaction effect on FTOE values was also significant at both ages. Covariates in the model were postconceptional age (only at the neonatal age), SpO_2,_ ethnicity, and sex.

CHD = congenital heart disease; FTOE = fractional tissue oxygen extraction; SpO_2_ = preductal systemic oxygenation.

*p<0.05, **p≤0.001

**Supplemental Table 5. Effects of Cyanotic CHD on Cerebrovascular Stability at the Neonatal and 3-month Ages.**

|  | **Neonatal Age** | | | | | | |
| --- | --- | --- | --- | --- | --- | --- | --- |
| **rcSO_2_** | | **N=52** | **β** | **Standard Error** |  | **95% CI** | **p-value** |
| **Postconceptional Age (weeks)** | |  | -2.27 | 1.12 |  | (-4.52,  -0.02) | 0.05* |
| **Sex** | |  | -3.45 | 2.40 |  | (-8.29,1.38) | 0.16 |
| **Ethnicity** | |  | -0.71 | 1.79 |  | (-4.32,2.91) | 0.70 |
| **SpO_2_ (%)** | |  | 0.05 | 0.01 |  | (0.02,0.08) | ** |
| **Cyanosis** | |  |  |  |  |  |  |
| **No** | | 17 | Ref | - |  | - | - |
| **Yes** | | 35 | 9.86 | 2.53 |  | (4.76,14.95) | ** |
| **Posture** | |  |  |  |  |  |  |
| **Supine** | |  | Ref | - |  | - | - |
| **Sitting** | |  | 1.69 | 0.10 |  | (1.50,1.89) | ** |
| **Cyanosis-by-posture** | |  | 0.07 | 0.19 |  | (-0.44,0.30) | 0.72 |
|  | **3-Month Age** | | | | | | |
| **rcSO_2_** | | **N=45** | **β** | **Standard Error** |  | **95% CI** | **p-value** |
| **Sex** | |  | -3.22 | 2.76 |  | (-8.81, 2.36) | 0.25 |
| **Ethnicity** | |  | -0.69 | 1.77 |  | (-4.26, 2.89) | 0.70 |
| **SpO_2_ (%)** | |  | 0.31 | 0.02 |  | (0.28, 0.35) | ** |
| **Cyanosis** | |  |  |  |  |  |  |
| **No** | | 20 | Ref | - |  | - | - |
| **Yes** | | 25 | 2.20 | 2.83 |  | (-3.52, 7.92) | 0.44 |
| **Posture** | |  |  |  |  |  |  |
| **Supine** | |  | Ref | - |  | - | - |
| **Sitting** | |  | 1.88 | 0.15 |  | (1.58, 2.18) | ** |
| **Cyanosis-by-posture** | |  | -0.34 | 0.24 |  | (-0.14, 0.82) | 0.16 |

Mixed effects regression models for repeated measures assessed the associations of cyanotic vs. acyanotic defects and posture with rcSO_2_ values. The main effect of cyanosis tested our hypothesis of reduced cerebral oxygen saturation, and the cyanosis-by-posture interaction tested our hypothesis of decreased cerebrovascular stability in cyanotic CHD. We found that the main effects of cyanosis (versus acyanotic CHD) on rcSO_2_, while controlling for specified covariates, were both significant at the neonatal age but not at the 3-month age. The estimated cyanosis-by-posture interaction effect on rcSO_2_ values was not significant at either age. Covariates in the model were postconceptional age (only at the neonatal age), ethnicity, SpO_2,_ and sex.

CHD = congenital heart disease; rcSO_2_ = regional cerebral oxygenation; SpO_2_ = preductal systemic oxygenation

*p<0.05, **p≤0.001**Supplemental Table 6. Effects of Cyanotic vs. Acyanotic CHD on FTOE at the Neonatal and 3-month Ages.**

|  | **Neonatal Age** | | | | | | |
| --- | --- | --- | --- | --- | --- | --- | --- |
| **FTOE** | | **N=52** | **β** | **Standard Error** | | **95% CI** | **p-value** |
| **Postconceptional Age (weeks)** | |  | 0.023 | 0.012 | | (-0.001, 0.047) | 0.06 |
| **Sex** | |  | 0.036 | 0.027 | | (-0.018, 0.089) | 0.19 |
| **Ethnicity** | |  | 0.008 | 0.020 | | (-0.032, 0.048) | 0.70 |
| **SpO_2_ (%)** | |  | 0.008 | 0.000 | | (0.008, 0.008) | ** |
| **Cyanosis** | |  |  |  | |  |  |
| **Yes** | | 34 | Ref | - | | - | - |
| **No** | | 17 | -0.109 | 0.027 | | (-0.164, -0.054) | ** |
| **Posture** | |  |  |  | |  |  |
| **Supine** | |  | -0.020 | 0.001 | | (-0.022, -0.018) | ** |
| **Sitting** | |  | Ref | - | | - | - |
| **Cyanosis-by-posture** | |  | 0.003 | 0.002 | | (-0.022, -0.018) | ** |
|  | **3-Month Age** | | | | | | |
| **FTOE** | | **N=45** | **β** | | **Standard Error** | **95% CI** | **p-value** |
| **Sex** | |  | 0.038 | | 0.029 | (-0.021, 0.097) | 0.20 |
| **Ethnicity** | |  | 0.004 | | 0.019 | (-0.034, 0.043) | 0.82 |
| **SpO_2_ (%)** | |  | 0.004 | | 0.000 | (0.003, 0.004) | ** |
| **Cyanosis** | |  |  | |  |  |  |
| **Yes** | | 26 | Ref | | - | - | - |
| **No** | | 19 | -0.046 | | 0.031 | (-0.108, 0.016) | 0.02* |
| **Posture** | |  |  | |  |  |  |
| **Supine** | |  | -0.017 | | 0.002 | (-0.021, -0.013) | ** |
| **Sitting** | |  | Ref | | - | - | - |
| **Cyanosis-by-posture** | |  | 0.001 | | 0.003 | (-0.004, 0.007) | 0.62 |

Mixed effects regression models for repeated measures assessed the associations of cyanotic vs. acyanotic defects and posture with FTOE values. The main effect of cyanosis tested our hypothesis of reduced cerebral oxygen saturation, and the cyanosis-by-posture interaction tested our hypothesis of decreased cerebrovascular stability in cyanotic CHD. We found that the main effects of cyanosis (versus acyanotic CHD) on FTOE, while controlling for specified covariates, were both significant at the neonatal age but not at the 3-month age. However, the estimated cyanosis-by-posture interaction effect on FTOE values was not significant at either age. Covariates in the model were postconceptional age (only at the neonatal age), ethnicity, SpO_2,_ and sex.

CHD = congenital heart disease; FTOE = fractional tissue oxygen extraction; SpO_2_ = preductal systemic oxygenation

*p<0.05, **p≤0.001
